# Supplementary material for: Validity of the Central Sensitization Inventory to Address Human Assumed Central Sensitization: Newly Proposed Clinically Relevant Values and Associations
Source: J Clin Med. 2023 Jul 23;12(14):4849. doi: 10.3390/jcm12144849 (PMC10381378; doi:10.3390/jcm12144849)
Supplement: Supplementary file 1 [file jcm-12-04849-s001.zip › CSI V4.0 - Supplement A.pdf]

Supplement A: Table S1. CSI cut-off scores, comparing CSS+ (N=1730) and healthy volunteers (N=250); comparing female CSS+ (N=1213) and female healthy volunteers (N=157); comparing male CSS+ (N=517) and male healthy volunteers (N=93)

	All (AUC: 0.953)			***Women (AUC: 0.956)***			***Men (AUC: 0.947)***		
Cut-off scores	Sensitivity	Specificity	Youden Index	Sensitivity	Specificity	Youden Index	Sensitivity	Specificity	Youden Index
19							0.9536	0.7419	0.6955
20	0.9613	0.6680	0.6293	0.9687	0.6115	0.5801	0.9439	0.7634	0.7073
21	0.9584	0.7240	0.6824	0.9662	0.6752	0.6414	0.9400	0.8065	0.7465
22	0.9462	0.7360	0.6822	0.9547	0.6879	0.6426	0.9265	0.8172	0.7437
23	0.9387	0.7720	0.7107	0.9505	0.7325	0.6830	0.9110	0.8387	0.7497
24	0.9324	0.7920	0.7244	0.9431	0.7516	0.6947	0.9072	0.8602	0.7674
25	0.9214	0.8280	0.7494	0.9349	0.7771	0.7119	**0.8897**	**0.9140**	**0.8037**
26	0.9127	0.8480	0.7607	0.9291	0.8089	0.7380	0.8743	0.9140	0.7883
27	0.8994	0.8680	0.7674	0.9176	0.8408	0.7583	0.8569	0.9140	0.7708
28	0.8855	0.8920	0.7775	0.9052	0.8726	0.7778	0.8395	0.9247	0.7642
29	0.8705	0.9080	0.7785	0.8928	0.8854	0.7782	0.8182	0.9462	0.7644
30	**0.8538**	**0.9280**	**0.7818**	0.8805	0.9108	0.7913	0.7911	0.9570	0.7481
31	0.8341	0.9360	0.7701	0.8615	0.9236	0.7851	0.7698	0.9570	0.7268
32	0.8208	0.9440	0.7648	0.8483	0.9299	0.7782	0.7563	0.9677	0.7240
33	0.7988	0.9680	0.7668	**0.8252**	**0.9682**	**0.7934**	0.7369	0.9677	0.7047
34	0.7792	0.9680	0.7472	0.8054	0.9682	0.7736	0.7176	0.9677	0.6853
35	0.7572	0.9680	0.7252	0.7824	0.9682	0.7505	0.6983	0.9677	0.6660
36	0.7289	0.9720	0.7009	0.7527	0.9682	0.7208	0.6731	0.9785	0.6516
37	0.7087	0.9720	0.6807	0.7329	0.9682	0.7010	0.6518	0.9785	0.6303
38	0.6855	0.9720	0.6575	0.7073	0.9682	0.6755	0.6344	0.9785	0.6129
39	0.6618	0.9840	0.6458	0.6859	0.9873	0.6732	0.6054	0.9785	0.5839
40	0.6399	0.9880	0.6279	0.6653	0.9936	0.6589	0.5803	0.9785	0.5588
41	0.6168	0.9920	0.6088	0.6406	0.9936	0.6342	0.5609	0.9892	0.5502
42	0.5931	0.9920	0.5851	0.6183	0.9936	0.6119	0.5338	0.9892	0.5231

Abbreviations: CSI: central sensitization inventory; CSS: central sensitivity syndromes; AUC: area under the curve.

Statistics: ROC analyses were used to calculate AUC, sensitivity and specificity, and the Youden Index.
