# Supplementary material for: Validity of the Central Sensitization Inventory to Address Human Assumed Central Sensitization: Newly Proposed Clinically Relevant Values and Associations
Source: J Clin Med. 2023 Jul 23;12(14):4849. doi: 10.3390/jcm12144849 (PMC10381378; doi:10.3390/jcm12144849)
Supplement: Supplementary file 1 [file jcm-12-04849-s001.zip › CSI V4.0 - Supplement C.pdf]

**Supplement C: Table S2. Multiple backward linear regression analysis on influencing factors of the CSI score, including 1537 patients with chronic pain (898 women and 639 men)**

***Total group***

| *Model* | Factor | β | p-value |
|---|---|---|---|
| *1 (Adjusted $R^2$ 0.577)* | (Constant) | 69.708 | <0.001 |
| | Sex | 2.788 | <0.001 |
| | Age | -0.067 | <0.001 |
| | BMI | 0.103 | 0.035 |
| | Pain severity | -0.363 | 0.047 |
| | Pain catastrophizing | 0.160 | <0.001 |
| | Pain disability | 0.131 | <0.001 |
| | Number of pain locations | 2.175 | <0.001 |
| | Number of reported CCSs | 3.244 | <0.001 |
| | Physical component SF 12 (quality of life) | -0.400 | <0.001 |
| | Mental component SF-12 (quality of life) | -0.729 | <0.001 |

***Women***

| Model | Factor | β | p-value |
|---|---|---|---|
| *1 (Adjusted $R^2$ 0.576)* | (Constant) | 80.447 | <0.001 |
| | Age | -0.099 | <0.001 |
| | BMI | 0.125 | 0.034 |
| | **Pain severity** | -0.310 | 0.182 |
| | Pain catastrophizing | 0.099 | 0.005 |
| | Pain disability | 0.121 | <0.001 |
| | Number of pain locations | 2.240 | <0.001 |
| | Number of reported CCSs | 3.210 | <0.001 |
| | Physical component SF 12 (quality of life) | -0.393 | <0.001 |
| | Mental component SF-12 (quality of life) | -0.801 | <0.001 |
| *2 (Adjusted $R^2$ 0.576)* | (Constant) | 79.434 | <0.001 |
| | Age | -0.099 | <0.001 |
| | BMI | 0.128 | 0.029 |
| | Pain catastrophizing | 0.091 | 0.009 |
| | Pain disability | 0.101 | 0.001 |
| | Number of pain locations | 2.236 | <0.001 |
| | Number of reported CCSs | 3.211 | <0.001 |
| | Physical component SF 12 (quality of life) | -0.395 | <0.001 |
| | Mental component SF-12 (quality of life) | -0.802 | <0.001 |

***Men***

| Model | Factor | β | p-value |
|---|---|---|---|
| *1 (Adjusted $R^2$ 0.546)* | (Constant) | 65.824 | <0.001 |
| | **Age** | -0.006 | 0.840 |
| | BMI | 0.034 | 0.700 |
| | Pain severity | -0.517 | 0.078 |
| | Pain catastrophizing | 0.255 | <0.001 |
| | Pain disability | 0.153 | <0.001 |
| | Number of pain locations | 2.037 | <0.001 |
| | Number of reported CCSs | 3.241 | <0.001 |
| | Physical component SF 12 (quality of life) | -0.436 | <0.001 |
| | Mental component SF-12 (quality of life) | -0.621 | <0.001 |
| *2 (Adjusted $R^2$ 0.546)* | (Constant) | 65.563 | <0.001 |
| | **BMI** | 0.032 | 0.717 |
| | Pain severity | -0.518 | 0.077 |
| | Pain catastrophizing | 0.255 | <0.001 |
| | Pain disability | 0.153 | <0.001 |
| | Number of pain locations | 2.043 | <0.001 |
| | Number of reported CCSs | 3.237 | <0.001 |
| | Physical component SF 12 (quality of life) | -0.434 | <0.001 |
| | Mental component SF-12 (quality of life) | -0.622 | <0.001 |
| *3 (Adjusted $R^2$ 0.547)* | (Constant) | 66.647 | <0.001 |
| | **Pain severity** | -0.517 | 0.077 |
| | Pain catastrophizing | 0.256 | <0.001 |
| | Pain disability | 0.153 | <0.001 |
| | Number of pain locations | 2.039 | <0.001 |
| | Number of reported CCSs | 3.235 | <0.001 |
| | Physical component SF 12 (quality of life) | -0.438 | <0.001 |
| | Mental component SF-12 (quality of life) | -0.622 | <0.001 |
| *4 (Adjusted $R^2$ 0.545)* | (Constant) | 65.378 | <0.001 |
| | Pain catastrophizing | 0.234 | <0.001 |
| | Pain disability | 0.123 | 0.001 |
| | Number of pain locations | 2.048 | <0.001 |
| | Number of reported CCSs | 3.228 | <0.001 |
| | Physical component SF 12 (quality of life) | -0.444 | <0.001 |
| | Mental component SF-12 (quality of life) | -0.629 | <0.001 |

**Abbreviations**: *CSI: central sensitization inventory; β: unstandardized beta; BMI: body mass index; CSSs: central sensitivity syndromes; SF12: short-form 12-item health questionnaire.*

**Statistics:** *Multiple regression analysis (backwards); p-value significant <0.05*
