# Supplementary material for: Validity of the Central Sensitization Inventory to Address Human Assumed Central Sensitization: Newly Proposed Clinically Relevant Values and Associations
Source: J Clin Med. 2023 Jul 23;12(14):4849. doi: 10.3390/jcm12144849 (PMC10381378; doi:10.3390/jcm12144849)
Supplement: Supplementary file 1 [file jcm-12-04849-s001.zip › CSI V4.0- Supplement B.pdf]

Supplement B: Figure S1. ROC curves all patients, women and men CSS+ vs healthy volunteers.

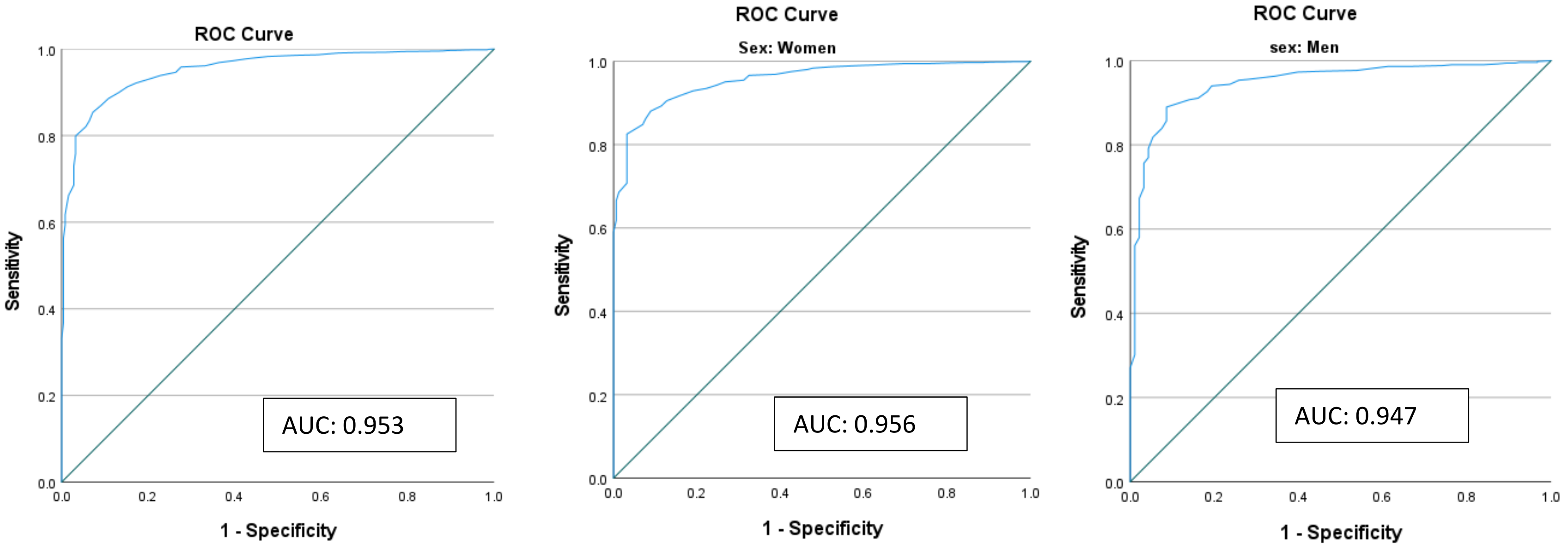

Abbreviations: *CSS: central sensitivity syndromes; AUC: Area Under the Curve*
